# Securinine from *Phyllanthus glaucus* Induces Cell Cycle Arrest and Apoptosis in Human Cervical Cancer HeLa Cells

**DOI:** 10.1371/journal.pone.0165372

**Published:** 2016-10-28

**Authors:** Justyna Stefanowicz-Hajduk, Barbara Sparzak-Stefanowska, Mirosława Krauze-Baranowska, J. Renata Ochocka

**Affiliations:** 1 Department of Biology and Pharmaceutical Botany, Medical University of Gdańsk, Gdańsk, Poland; 2 Department of Pharmacognosy with Medicinal Plant Garden, Medical University of Gdańsk, Gdańsk, Poland; University of PECS Medical School, HUNGARY

## Abstract

**Background:**

The *Securinega*-type alkaloids occur in plants belonging to *Euphorbiaceae* family. One of the most widely distributed alkaloid of this group is securinine, which was identified next to allosecurinine in *Phyllanthus glaucus* (leafflower). Recently, some *Securinega*-type alkaloids have paid attention to its antiproliferative potency towards different cancer cells. However, the cytotoxic properties of allosecurinine have not yet been evaluated.

**Methods:**

The cytotoxicity of the extract, alkaloid fraction obtained from *P*. *glaucus*, isolated securinine and allosecurinine against HeLa cells was evaluated by real-time xCELLigence system and 3-(4,5-dimethylthiazol-2-yl)-2,5-diphenyltetrazolium bromide (MTT) assay. Apoptosis was detected by annexin V and 7-amino-actinomycin (7-AAD) staining and confirmed with fluorescent Hoechst 33342 dye. The assessment of mitochondrial membrane potential (MMP), reactive oxygen species (ROS) generation, the level of extracellular signal-regulated protein kinases 1 and 2 (ERK1/2), caspase-3/7 activity and cell cycle analysis were measured by flow cytometry. The enzymatic activity of caspase-9 was assessed by a luminometric assay. The expression of apoptosis associated genes was analyzed by real-time PCR.

**Results:**

The experimental data revealed that securinine and the alkaloid fraction were significantly potent on HeLa cells growth inhibition with IC_50_ values of 7.02 ± 0.52 μg/ml (32.3 μM) and 25.46 ± 1.79 μg/ml, respectively. The activity of allosecurinine and *Phyllanthus* extract were much lower. Furthermore, our study showed that the most active securinine induced apoptosis in a dose-dependent manner in the tested cells, increased the percentage of ROS positive cells and depolarized cells as well as stimulated the activity of ERK1/2, caspase-9 and -3/7. Securinine also induced cell cycle arrest in S phase. Real-time PCR analysis showed high expression of TNFRSF genes in the cells stimulated with securinine.

**Conclusions:**

Securinine induces apoptosis and activates cell cycle checkpoints in HeLa cells which is associated with oxidative stress. The results indicate that the mitochondrial pathway is involved in the programmed cell death.

## Introduction

**Fig 1:**
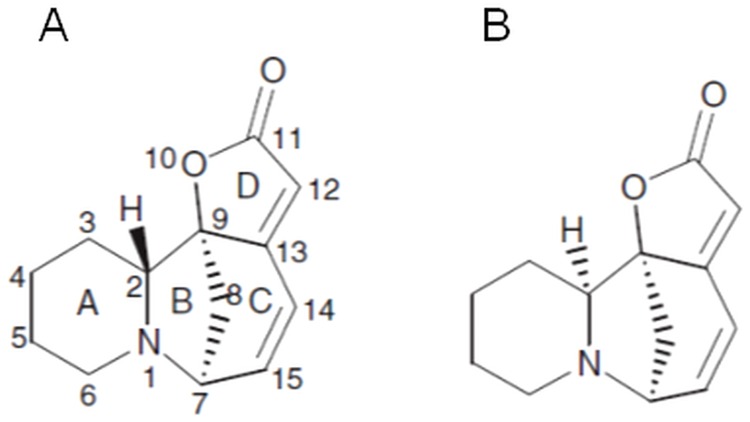
Structure of securinine (A) and allosecurinine (B).

The *Phyllanthus* species (leafflowers) (*Euphorbiaceae*) are ones of the most widely distributed plants throughout the Amazon rainforest as well as other tropical and subtropical regions [1]. Over the years plants from the genus *Phyllanthus* have gained reputation in folk and traditional medicine for numerous healing properties which were confirmed in studies of pharmacological activity, like antiviral activity against *hepatitis B*, antihepatotoxic and liver-protecting activity, as well as antitumor and anticarcinogenic properties [1, 2]. Leafflowers produce a diverse array of bioactive compounds, with lignans as one of the major groups of secondary metabolites next to flavonoids, ellagotannins and *Securinega*-type alkaloids [1]. These alkaloids are a class of rare, natural compounds initially discovered in leaves of *Securinega suffruticosa*. Later they were recognized in other plants of *Euphorbiaceae*–*Flueggea* and *Phyllanthus* species [3]. The occurrence of *Securinega*-type alkaloids in leafflowers is relatively narrow and until now they have been confirmed only in a few *Phyllanthus* species, including *P*. *amarus*, *P*. *niruri*, *P*. *discoideus*, *P*. *niruroides* or *P*. *simplex* [1]. Recent research has demonstrated that *in vitro* shoot culture of *Phyllanthus glaucus* Wall. ex Müll. Arg. is a source of *Securinega*-type alkaloids with securinine and its isomer allosecurinine (Fig 1) as dominating compounds in alkaloid fraction [4].

*Phyllanthus glaucus* is a deciduous shrub growing at elevation of 200–1000 m a. s. l. The species is mainly found in the central parts of China. The roots of *P*. *glaucus* were used medicinally for the treatment of rheumatoid arthritis and malnutrition in children by the local people of its growing areas [5]. Securinine has been used clinically in several countries in the treatment of amyotrophic lateral sclerosis, poliomyelitis and multiple sclerosis [3] and that is related to well-established function of the compound as a GABA receptor antagonist [6].

The research conducted during last two decades paid attention to cytotoxic activity of securinine and other *Securinega*-type alkaloids. It was found that securinine and virosecurinine induce apoptosis in several cancer cell lines—human breast cancer cells MCF-7 [7], human promyelocytic leukemia cells HL-60 [8], human erythroleukemic cells K-562 [9], human colon cancer cells HCT 116 [10] and SW480 [11, 12]. By now the cytotoxic potential of allosecurinine has not been evaluated. Moreover, the cytotoxic activity of *Securinega*-type alkaloids against HeLa cell line remains unknown as well.

As a part of a search for novel activities of *Phyllanthus* species our paper concerns evaluation of cytotoxic activity of *P*. *glaucus* extract and its alkaloid constituents on human cervical cancer cells (HeLa). Securinine was also investigated towards mechanisms that play a role in inhibition of HeLa cells proliferation and induction of apoptosis.

## Materials and Methods

### Plant in vitro culture conditions

The reagents used for culture medium preparation were from Sigma-Aldrich (Sigma-Aldrich, St. Louis, MO, USA). Water was produced using Millipore system (Molsheim, France). The culture medium was supplemented with 3.0% w/v sucrose and was solidified with 0.7% w/v agar. The pH was adjusted to 5.8 prior to autoclaving (0.1 MPa, 121°C, 21 min). The culture was maintained in a growth chamber at 24±1°C, under a 16 h (light)/8 h (dark) photoperiod (white fluorescent lamps, 36W, light intensity 88± 8 μmol m−2 s−1, Philips, Amsterdam, The Netherlands).

### Plant material and explants preparation

The seeds used for development of *in vitro* cultures of *P*. *glaucus* originated from the Botanical Garden in Shanghai (China). The seeds were obtained within the framework of plant material exchange between the Medicinal Plant Garden of the Medical University of Gdańsk and the mentioned above botanical garden. The species was identified by specialists in botany and the voucher specimen is kept in the herbarium of the Medicinal Plant Garden of the Medical University of Gdańsk (Poland).

Before germination, the seeds were pre-washed with 1% commercial detergent for 1 min, and washed with water (0.5 h) followed by 1 min treatment with 70% *v*/*v* aqueous ethanol. The main sterilization was conducted with sodium hypochlorite (10% solution of commercial bleach “Domestos”, Unilever Polska, Warszawa, Poland) for 30 min. The seeds were rinsed three times with sterile water (2x15 min, 1x30 min), placed into petri dishes lined with wet filtration paper, and held in the dark at 24±1°C. After seeds germination the dishes were moved to a growth chamber on the Murashige and Skoog (MS) medium [13] without plant growth regulators (PGRs). After six weeks plantlets were cut into nodal section and moved to the MS medium supplemented with BAP (benzylaminopurine) 0.5 mg/l and IBA (indole-3-butyric acid) 0.5 mg/l. The shoots were subcultured in 5-weeks intervals. The collected plant material was lyophilized and pulverized.

### Preparation of dry extract and its phytochemical analysis for the studies of cytotoxic activity

The shoots of *P*. *glaucus* (2 g) harvested on MS medium supplemented with BAP 0.5 mg/l and IBA 0.5 mg/l were extracted with methanol in boiling temperature (3×150 ml, 3×30 min). The obtained extracts were combined, filtrated and reduced under reflux condenser. The reduced methanol extract was mixed with water and lyophilized.

Extraction of alkaloid fraction, isolation of securinine and allosecurinine and quantitative analyses comprising flavan-3-ol derivatives, sterols and determination of triterpenes were performed according to previously established methods [4, 14, 15]. The lyophilized extract and alkaloid fraction [4] were dissolved in absolute methanol at concentration of 2 mg/ml. The isolated compounds were dissolved in dimethyl sulfoxide (DMSO) at concentration of 10 mg/ml prior cytotoxic activity determination.

### Cell line culture

The human cervical adenocarcinoma cell line (HeLa S3) was obtained from the American Type Culture Collection (ATCC, USA). The cell line was cultured in Dulbecco’s Modified Eagle’s Medium (DMEM) supplemented with 10% (v/v) fetal bovine serum (FBS), 2 mM L-glutamine, 100 units/ml of penicillin, 100 μg/ml of streptomycin (Sigma-Aldrich), and was kept at 37°C in a humidified 5% CO_2_ incubator.

### MTT assay

The viability of the cells was determined by 3-(4,5-dimethylthiazol-2-yl)-2,5-diphenyltetrazolium bromide (MTT) assay. HeLa cells were seeded in 96-well plates at a density of 5x10^3^ cells/well and treated for 24 h with the extract, alkaloid fraction and allosecurinine at final concentrations of 5.0–100.0 μg/ml, respectively. The cells were exposed to securinine in the concentration range of 1.0–20.0 μg/ml. The maximal concentrations of the solvents used in all the MTT experiments were 5.0% (v/v) and 1.0% (v/v) for methanol and DMSO, respectively. Following treatment, the cells were prepared as described previously [16]. The absorption of the obtained formazan solution was measured with a plate reader (Epoch, BioTek Instruments, USA). The viability results are presented as IC_50_ mean values of at least three independent experiments.

### RTCA cell proliferation assay

The xCELLigence Real-Time Cell Analyzer Dual Plate (RTCA DP, ACEA Biosciences, USA) was used for monitoring of viability of HeLa cells treated with the extract, alkaloid fraction, allosecurinine and securinine for 24 h. The cells were seeded at a density of 2x10^4^/well into E-plate 16 (ACEA Biosciences). When the cells entered log phase, the extract, fraction and allosecurinine were added in the final concentration range of 5.0–100.0 μg/ml, respectively. Securinine was tested at the concentrations of 1.0–20.0 μg/ml. The maximal concentrations of the extract/fraction/compounds solvents used in the RTCA experiments—methanol and DMSO did not exceed 5.0% (v/v) and 1.0% (v/v), respectively. To obtain viability profiles and calculate IC_50_ values, we used the RTCA software v. 1.2.1. All the experiments were performed in duplicate, in three independent repeats.

### Hoechst staining

The apoptotic effect of securinine was analyzed by the blue fluorescent Hoechst 33342 dye (Life Technologies, USA). HeLa cells were seeded in 12-well plates at a density of 1x10^5^/well. The cells were treated with securinine in the final concentration range of 1.0–50.0 μg/ml for 24 h. The concentration of securinine solvent (DMSO) did not exceed 0.5% (v/v). After 24 h, the cells were stained according to previously described protocol [16] and observed under a fluorescent microscope (Leica, Switzerland).

### Apoptosis assay

To estimate the apoptotic effect of securinine on HeLa cells, we performed experiments using Annexin V and Dead Cell Assay (Merck Millipore, Germany). The cells were seeded in 12-well plates at a density of 1x10^5^/well and treated with securinine at concentrations of 1.0–50.0 μg/ml. The securinine solvent—DMSO was added to the control cells at a concentration of 0.5% (v/v). After treating, the cells were harvested, then stained with annexin V/phycoerythrin and 7-amino-actinomycin (7-AAD) and analyzed by Muse Cell Analyzer (Merck Millipore), following the protocol provided by the manufacturer. The experiments were performed at least in three independent repeats.

### Caspase-9 and -3/7 activity

The cells were seeded in 12-well plates (1x10^5^/well) and treated with securinine at concentrations of 1.0–50.0 μg/ml. The control cells were exposed to DMSO at a concentration of 0.5% (v/v). After 6 h and 24 h of exposure, the activity of caspase-9 was measured by Caspase-Glo 9 Assay Kit (Promega, USA) and Glomax Multi+ Detection System (Promega), according to the manufacturer’s instruction. The activity of caspase-3/7 was assessed after 24 h of exposure the cells to securinine. Then the cells were harvested and prepared using Muse Caspase-3/7 Assay Kit (Merck Millipore) according with the manufacturer’s protocol. The stained cells were analyzed by Muse Cell Analyzer. The experiments were performed at least in three independent repeats.

### Assessment of mitochondrial membrane potential (MMP)

Hela cells were seeded at a density of 1x10^5^ cells/well and treated with securinine at concentrations of 1.0–50.0 μg/ml. The concentration of DMSO as a control sample did not exceed 0.5% (v/v). After 5 h and 8 h of exposure, the cells were harvested and prepared using Muse MitoPotential Assay Kit (Merck Millipore) according with the manufacturer’s protocol. The percentage of depolarized/live cells was determined by Muse Cell Analyzer. All the experiments were independently repeated at least three times.

### Measurement of reactive oxygen species (ROS) generation

To determine the percentage of HeLa cells undergoing oxidative stress, we treated the cells (1x10^5^/well) with securinine at concentrations of 1.0–50.0 μg/ml. The concentration of DMSO added to the cells (control sample) did not exceed 0.5% (v/v). After 24 h of exposure, the cells were stained with Muse Oxidative Stress Kit (Merck Millipore), following the manufacturer’s protocol and analyzed by Muse Cell Analyzer. The results were obtained at least in three independently repeated experiments.

### Assessment of ERK1/2 activation

HeLa cells were seeded at a density of 1x10^5^ cells/well, treated with securinine at concentrations of 1.0–50.0 μg/ml. The concentration of DMSO as a control sample did not exceed 0.5% (v/v). After 24 h and 48 h of exposure, the cells were harvested and prepared with Muse MAPK Activation Dual Detection Kit (Merck Millipore), following the manufacturer’s protocol. The percentage of the ERK1/2 activated cells in population after securinine stimulation was analyzed by Muse Cell Analyzer. All the experiments were independently repeated at least three times.

### Cell cycle analysis

HeLa cells were seeded (5x10^5^/well) and incubated with securinine at concentrations of 10.0 and 20.0 μg/ml for 24 h and 48 h. DMSO concentration added to the cells was 0.2% (v/v). After treating, the cells were harvested and stained with reagents of Muse Cell Cycle Assay Kit (Merck Millipore). Quantitative measurement of the percentage of the cells in G0/G1, S, and G2/M phases of cell cycle was done on Muse Cell Analyzer. The experiments were performed in triplicate.

### Real-time PCR

HeLa cells were seeded (3x10^6^) and treated with DMSO and securinine at a concentration of 0.1% (v/v) and 10.0 μg/ml, respectively for 24 h. The isolation of total RNA was performed with RNeasy Mini Kit (Qiagen, The Netherlands) following the manufacturer’s instructions. The concentration of isolated RNA was measured and cDNA synthesis was performed using Maxima First Strand cDNA Synthesis Kit (Thermo Scientific, USA), according with the manufacturer’s protocol.

Real-time PCR gene expression arrays. cDNA obtained from RNA of the cells, was applied on The Applied Biosystems TaqMan Array Human Apoptosis 96-well FAST Plates (Life Technologies, USA). Each plate contains 88 assays for apoptosis associated genes and 4 assays for candidate endogenous control genes (Table A in S1 File). The PCR reactions were set according to the manufacturer’s instructions and performed on StepOnePlus Real-Time PCR System (Life Technologies). The resulting data were analyzed with StepOne software v2.3. based on the comparative dCT method.

### Statistical analysis

All data are expressed as mean values ± standard deviation (SD). Statistical comparisons of the results were evaluated using the Student’s t-test.

## Results

### Phytochemical analysis of *P*. *glaucus* extract

*Phyllanthus glaucus* shoots obtained *in vitro* were investigated in terms of alkaloids [4], flavan-3-ol derivatives, sterol and triterpenes. Using quantitative HPTLC method, four flavan-3-ol derivatives, (–)-epicatechin, (+)-catechin, (–)-epigallocatechin, and (–)-gallocatechin, were identified. The determined content of (-)-gallocatechin was 0.47 mg/g d.w. (dry weight). The concentration of other identified catechins was below the LOQ (limit of quantification). Additionally, in the study of sterols and triterpenes the presence of β-sitosterol was detected (3.33 mg/g d. w.) and the traces of β-amyrin. The determination of *Securinega*-type alkaloids was described in previous study [4].

### Securinega-type alkaloids decreased HeLa cells viability

The effect of *Phyllanthus* extract, alkaloid fraction and selected alkaloids—allosecurinine and securinine on the cells viability was examined by xCELLigence system. This platform allows detection of cellular processes by measuring the electronic impedance of sensor-electrodes located at the bottom of wells in the RTCA plates. Changes in the electronic signals of the electrodes reflect changes in cell number, viability, morphology and cytoskeletal dynamics. This effect is described by a parameter called Cell Index (CI) which enables to calculate the IC_50_ value in every point of time during an experiment [17, 18].

HeLa cells were exposed to different concentrations of the *Phyllathus* extract, alkaloid fraction, allosecurinine and securinine. The results obtained during the experiments showed that securinine and the fraction were significantly more active to the cells than allosecurinine and the extract with IC_50_ values of 7.02 ± 0.52 μg/ml (32.3 μM), 25.46 ± 1.79 μg/ml, 52.88 ± 4.62 μg/ml (243.4 μM) and above 100.0 μg/ml, respectively (Table 1).

**Table 1:** IC_50_ values (μg/ml) of the *Phyllanthus* compounds, fraction and extract based on the xCELLigence system (RTCA) and MTT assay.

Extract, alkaloid fraction and pure compounds	IC_50_ [μg/ml]	
	RTCA	MTT assay
Securinine	7.02 ± 0.52; R^2^* = 0.99	9.84 ± 0.83
Allosecurinine	52.88 ± 4.62; R^2^ = 0.90	48.11 ± 2.51
Alkaloid fraction	25.46 ± 1.79; R^2^ = 0.99	26.44 ± 3.08
Extract	>100	>100

*R^2^ –the coefficient of determination

**Fig 2:**
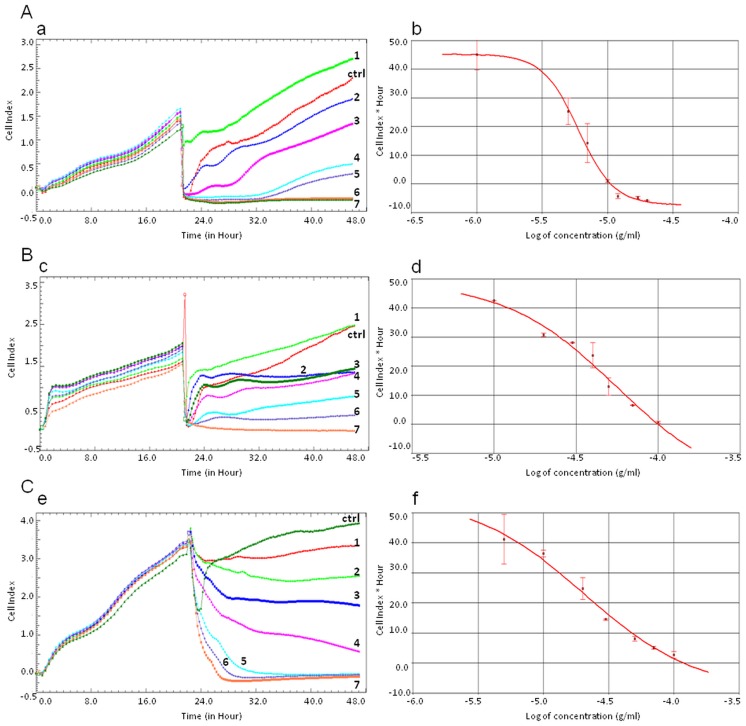
RTCA measurement of Cell Index values during HeLa cells incubation with *Securinega*-type alkaloids (A, B) and the alkaloid fraction (C). The numeric labels on the curves (a, c, e) represent the increasing concentration values of securinine (A) (1.0, 5.0, 7.0, 10.0, 12.0, 17.0, 20.0 μg/ml, respectively), allosecurinine (B) (10.0, 20.0, 30.0, 40.0, 50.0, 70.0, 100.0 μg/ml, respectively) and the alkaloid fraction (C) (5.0, 10.0, 20.0, 30.0, 50.0, 70.0, 100.0 μg/ml, respectively). The cells treated with 1.0% DMSO (A, B) and 5.0% MeOH (C) were the control samples (ctrl). The IC_50_ values of securinine (b), allosecurinine (d) and the alkaloid fraction (f) were calculated based on the dose-response curves of the cell index by the xCELLigence system. Error bars represent standard deviations.

The RTCA profiles of the cells proliferation showed that the studied compounds (Fig 2A and 2B) as well as the analyzed fraction showed a dose-dependent and time-dependent activity (Fig 2C).

To confirm the results from the RTCA experiments, we performed MTT assay using the same concentration range of the *Phyllanthus* compounds, alkaloid fraction and extract. The IC_50_ data obtained in these experiments were 9.84 ± 0.83 μg/ml (45.3 μM), 26.44 ± 3.08 μg/ml, 48.11 ± 2.51 μg/ml (221.5 μM) and above 100.0 μg/ml for securinine, the alkaloid fraction, allosecurinine and the extract, respectively (Table 1).

### Effect of securinine-induced cell apoptosis

**Fig 3:**
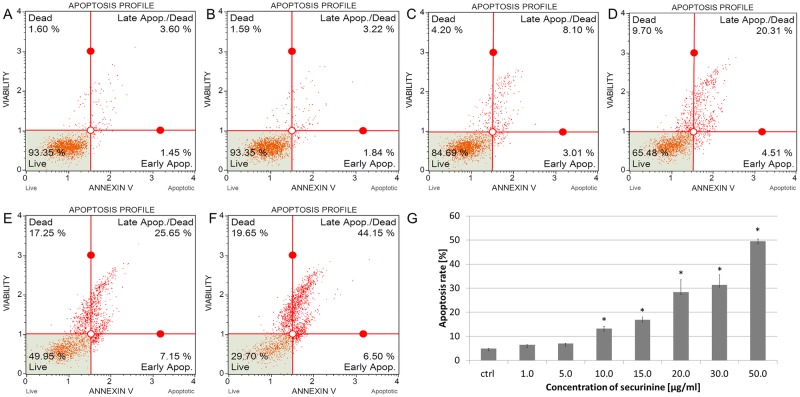
Securinine induced apoptosis in HeLa cells. The distribution of the apoptotic cells was determined by flow cytometry. The cells were incubated with 0.5% DMSO (A) and securinine at concentrations of 1.0 (B), 5.0, 10.0 (C), 15.0, 20.0 (D), 30.0 (E) and 50.0 μg/ml (F) for 24 h. The percentage of early and late apoptotic cells (apoptosis rate) was estimated in comparison to the DMSO control (G). Each sample was run at least in triplicate. Error bars represent standard deviations. Significant differences relative to the control are marked with an “*” (p<0.05).

The apoptotic effect of securinine was estimated after 24 h of treating the cells with the compound. The total percentage of early and late apoptotic cells (apoptosis rate) was 6.48 ± 0.04%, 7.05 ± 0.06%, 13.25 ± 0.99%, 16.91 ± 1.17%, 28.45 ± 5.12%, 31.40 ± 4.28% and 49.48 ± 1.01% for securinine concentrations of 1.0, 5.0, 10.0, 15.0, 20.0, 30.0 and 50.0 μg/ml, respectively (Fig 3). The obtained results indicated that the apoptotic effect of securinine increased in a dose-dependent manner.

**Fig 4:**
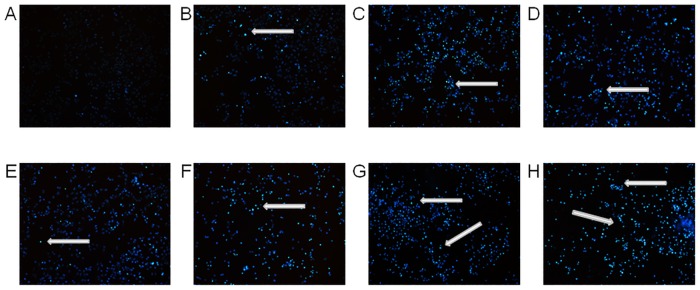
Securinine induced chromatin condensation in HeLa cells. The cells nuclei were stained with Hoechst 33342 dye after treating the cells with 0.5% DMSO (A) and securinine at concentrations of 1.0 (B), 5.0 (C), 10.0 (D), 15.0 (E), 20.0 (F), 30.0 (G) and 50.0 μg/ml (H). The cells exposed to securinine showed condensed chromatin in comparison to the control sample (DMSO). Arrows represent nuclear changes in the apoptotic cells.

To confirm induction of apoptosis and the changes in chromatin distribution within HeLa cells exposed to different concentrations of securinine (1.0–50.0 μg/ml), we stained the cells with Hoechst 33342 dye and visualized the cellular nuclei. The chromatin condensation and fragmentation of the cells nuclei were observed as compared to the control cells treated with DMSO at a concentration of 0.5% (v/v) (Fig 4).

### Securinine increased the activity of caspase-9 and -3/7 in HeLa cells

**Fig 5:**
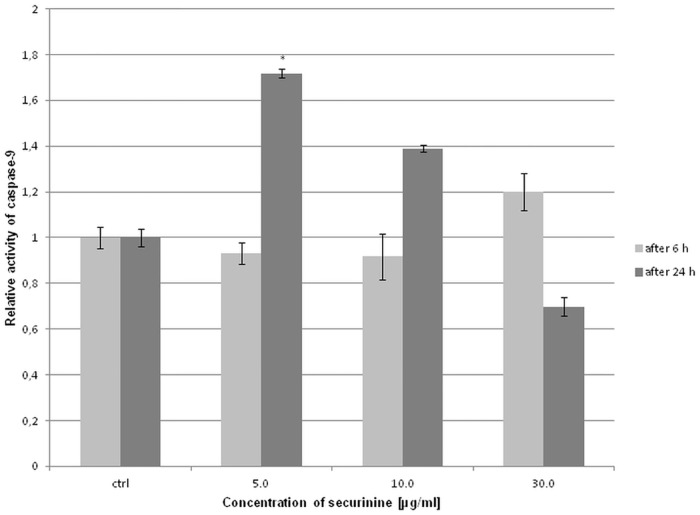
Securinine treatment changed the activity of caspase-9 in HeLa cells. The activity of the caspase was measured after 6 h and 24 h of incubating the cells with 0.5% DMSO and securinine at concentrations of 5.0, 10.0 and 30.0 μg/ml. Relative activity of caspase-9 was determined in comparison to the control (DMSO). Each sample was run at least in triplicate. Error bars represent standard deviations. Significant differences relative to the control are marked with an “*” (p<0.05).

The activity of caspase-9 was determined after 6 h and 24 h of treating the cells with securinine. We did not observe significant changes in the activity of caspase-9 after 6 h of treatment the cells with securinine. After 24 h, the obtained results show that the activity of caspase-9 significantly increased in the cells incubated with securinine concentration of 5.0 μg/ml (Fig 5).

**Fig 6:**
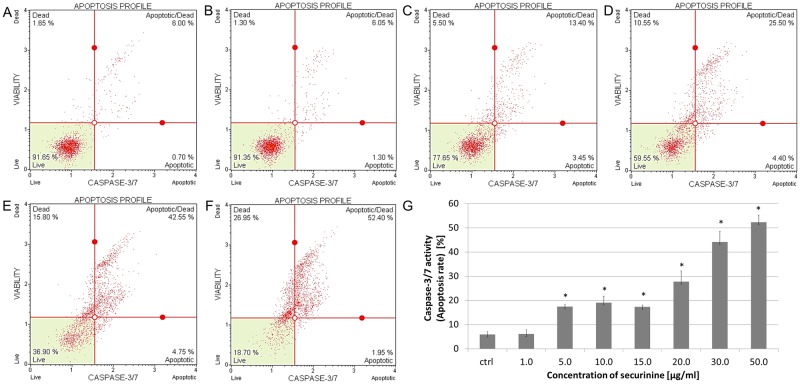
Securinine treatment increased activity of caspase-3/7 in HeLa cells. The activity of the caspase was measured after 24 h of incubating the cells with 0.5% DMSO (A) and securinine at concentrations of 1.0 (B), 5.0, 10.0 (C), 15.0, 20.0 (D), 30.0 (E) and 50.0 μg/ml (F). Caspase-3/7 activation is represented as the percentage of the apoptotic cells (apoptosis rate) which was determined in comparison to the control (DMSO) (G). Each sample was run at least in triplicate. Error bars represent standard deviations. Significant differences relative to the control are marked with an “*” (p<0.05).

To determine the percentage of the cells at various stages of apoptosis based on activity of executioner caspases, the cells were treated with different concentrations of securinine. The obtained cytometric results showed that securinine increased caspase-3/7 activity in a dose-dependent manner. The total percentage of apoptotic cells with activated caspase-3/7 was 6.13 ± 1.73%, 17.46 ± 0.74%, 19.11 ± 2.59%, 17.28 ± 0.75%, 27.71 ± 4.42%, 44.15 ± 4.45%, 52.33 ± 2.86% for securinine concentrations of 1.0, 5.0, 10.0, 15.0, 20.0, 30.0 and 50.0 μg/ml, respectively (Fig 6).

### Securinine modulated mitochondrial membrane potential (ΔΨm) in HeLa cells

**Fig 7:**
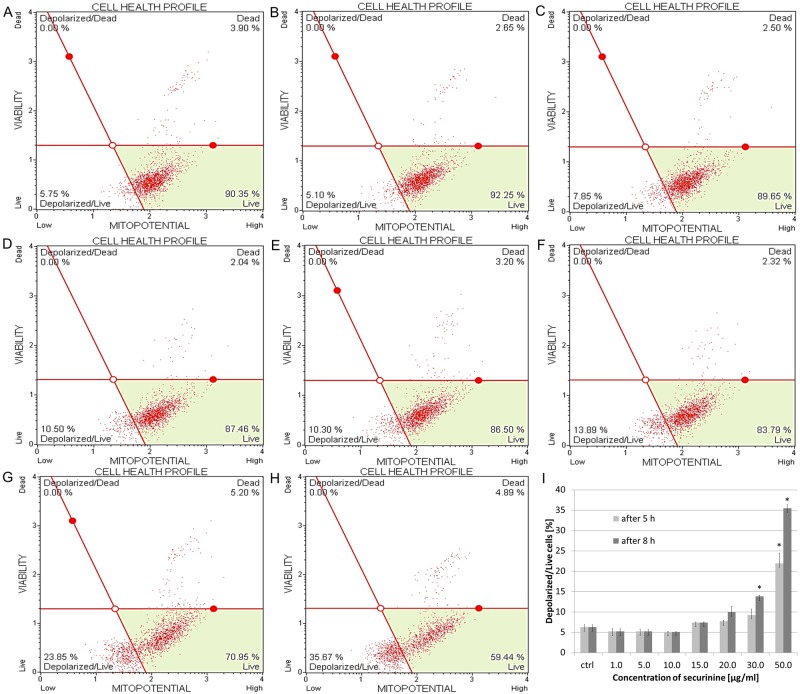
Securinine-induced changes in transmembrane mitochondrial potential in HeLa cells. The cells were exposed to 0.5% DMSO (A) and securinine at concentrations of 1.0, 5.0, 10.0 (B), 15.0, 20.0(C, D), 30.0 (E, F) and 50.0 μg/ml (G, H). The cells were treated for 5 h (A, B, C, E, G) and 8 h (A, B, D, F, H) and the extent of mitochondrial cell depolarization was determined in comparison to the DMSO control (I). Each sample was run at least in triplicate. Error bars represent standard deviations. Significant differences relative to the control are marked with an “*” (p<0.05).

HeLa cells were treated with securinine and the state of mitochondrial dysfunction of the cells was determined. Loss of the mitochondrial inner transmembrane potential is an indicator of cellular health and it is often observed in the early stages of apoptosis. The cells incubated with increasing concentrations of securinine demonstrated a decrease in fluorescence in comparison with the high fluorescence of the control cells (exposed to DMSO). The percentage of depolarized/live cells treated with the compound was 5.18 ± 0.74%, 5.18 ± 0.60%, 5.10 ± 0.07% and 7.38 ± 0.18% for securinine concentrations of 1.0, 5.0, 10.0, 15.0 μg/ml, respectively. These results related to both the incubating times in the experiment (5 h and 8 h). Above the concentration of 20.0 μg/ml, stronger dysfunction of mitochondrial membrane was observed after 8 h of treating the cells with the compound than after 5 h. The percentage of depolarized/live cells after 5 h of incubation was as follows: 7.68 ± 0.25%, 9.23 ± 1.46%, 21.95 ± 2.48% for securinine concentrations of 20.0, 30.0 and 50.0 μg/ml, respectively. After 8 h of incubation the obtained results were 9.93 ± 1.44%, 13.81 ± 0.22%, 35.46 ± 0.91% for securinine concentrations of 20.0, 30.0 and 50.0 μg/ml, respectively (Fig 7).

### Securinine increased ROS production in HeLa cells

**Fig 8:**
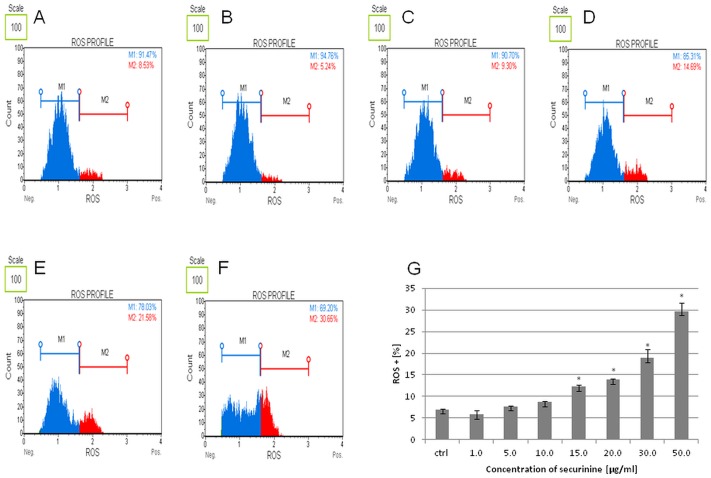
Securinine induced ROS production in HeLa cells. The cells were incubated with 0.5% DMSO (A) and securinine at concentrations of 1.0 (B), 5.0, 10.0 (C), 15.0, 20.0 (D), 30.0 (E) and 50.0 μg/ml (F) for 24 h. The extent of ROS production in the cells was determined in comparison to the control (DMSO) (G). Each sample was run at least in triplicate. Error bars represent standard deviations. Significant differences relative to the control are marked with an “*” (p<0.05).

ROS is defined as molecules playing a broad role in cell regulation and activation of signaling cascade and apoptosis. In our experiment, production of ROS was estimated in the cells after 24 h of treating them with securinine. Determination of the count and percentage of HeLa cells undergoing oxidative stress was based on the intracellular detection of superoxide radicals. We observed significant increase in ROS production for securinine concentrations of 30.0 and 50.0 μg/ml with 18.86 ± 2.08% and 29.75 ± 1.94% of the ROS (+) cells (cells exhibiting ROS), respectively after 24 h of incubation. For the other concentrations of the compound—1.0, 5.0, 10.0, 15.0 and 20.0 μg/ml the obtained results were 5.80 ± 1.02%, 7.73 ± 0.13%, 8.70 ± 0.25%, 12.23 ± 0.52% and 13.84 ± 0.35% of the ROS (+) cells, respectively (Fig 8).

### Securinine activated ERK1/2 proteins

ERK1/2 are involved in the evolutionarily conserved pathway that controls many cellular processes including cell proliferation, differentiation, survival and apoptosis. Regulation of the ERK pathway is associated with the dual-specificity kinases MEK1 and MEK2 which activate ERK1/2 through their phosphorylation [19]. In our study, we used two directly conjugated antibodies, a phospho-specific anti-phospho-ERK1/2 (Thr202/Tyr204, Thr185/Tyr187)-Phycoerythrin and an anti-ERK1/2-PECy5 to measure both the total and phosphorylated proteins.

**Fig 9:**
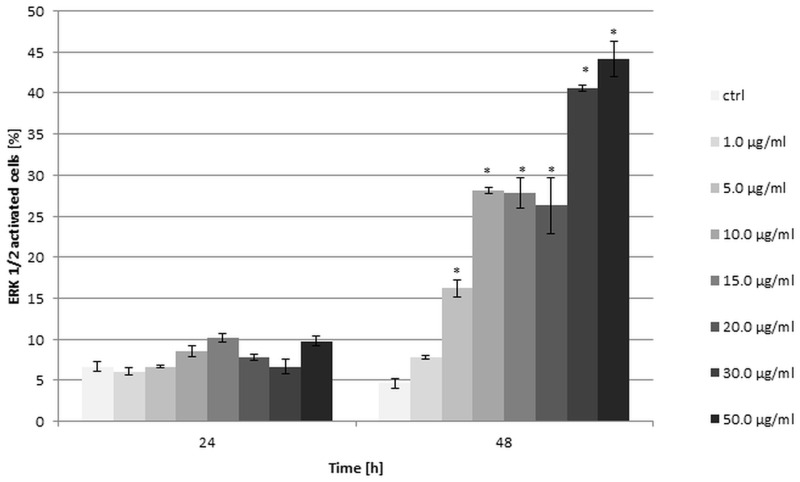
Securinine treatment increased ERK1/2 activity in HeLa cells. The cells were incubated with the compound at the concentrations of 1.0, 5.0, 10.0, 15.0, 20.0, 30.0 and 50.0 μg/ml for 24 h and 48 h. The percentage of ERK1/2 activated cells was measured by flow cytometry and determined in comparison to the control cells treated with 0.5% DMSO. Each sample was run in triplicate. Error bars represent standard deviations. Significant differences relative to the control are marked with an “*” (p<0.05).

The effect of securinine on the level of kinases ERK1/2 was assessed after 24 h and 48 h of incubating the cells with the tested compound. The results showed that the activity of ERK1/2 significantly changed after 48 h of the cells treating. The highest activity was observed for securinine concentrations of 30.0 and 50.0 μg/ml and the obtained values were 40.6 ± 0.42% and 44.15 ±2.19% of activated ERK1/2 cells, respectively. After 24 h, we did not observe significant changes in ERK1/2 activity for all the used concentrations of securinine in comparison with the control cells (Fig 9).

### Securinine induced cell cycle arrest in S phase

**Fig 10:**
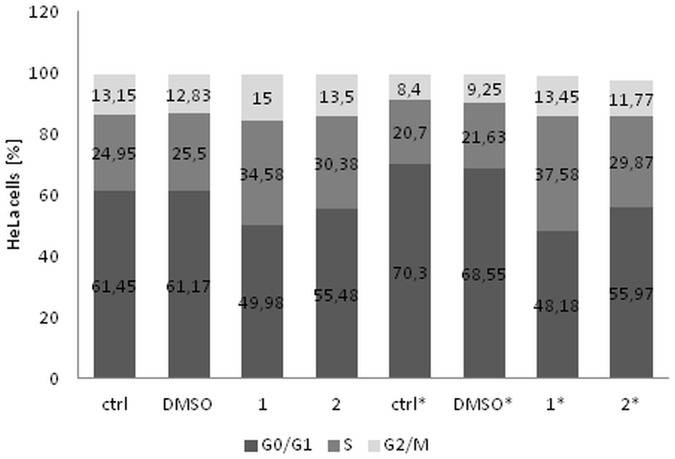
ecurinine treatment induced cell cycle arrest in S and G2/M phases in HeLa cells. The cells were stimulated with the compound at concentrations of 10.0 (1) and 20.0 μg/ml (2) and incubated for 24 h and 48 h (*). The percentage of the cells in each phases was measured by flow cytometry and determined in comparison to the untreated cells (ctrl) and DMSO control. Each sample was run in triplicate.

The effect of securinine on cell cycle distribution of HeLa cells was determined. The obtained results indicated that the compound arrested the cells in S and G2/M phases compared to the untreated cells (ctrl I) and cells incubated with the compound solvent (DMSO) (ctrl II). After 24 h of incubating, the S phase cells increased from 24.95% and 25.5% for ctrl I and ctrl II, respectively to 34.58% and 30.38% for securinine concentrations of 10.0 and 20.0 μg/ml, respectively. After 48 h, the percentage of the S phase cells treated with securinine was comparable to the results obtained after 24 h of incubation. However, in relating to the control cells the percentage of the S phase cells treated with securinine significantly increased after 48 h (Fig 10).

The increase in the amount of the cells in G2/M phase was stronger after 48 h then after 24 h of treating the cells with securinine in comparison to the controls. The obtained results were: 13.45% and 11.77% for 10.0 and 20.0 μg/ml of securinine, respectively comparing with 8.4% and 9.25% of the untreated cells and the cells treated with DMSO, respectively (Fig 10).

### Securinine increased expression of tumor necrosis factor receptor superfamily TNFRSF (TRAIL receptors) genes

To determine the extent of securinine on apoptotic pathways, the expression of 88 related apoptosis genes was tested with qPCR (qPCR human apoptosis array). The expression of genes with significant changes in comparison to the control was ≥2-fold over or ≤0.5-fold under the control (Figure A in S1 File). The highest expression we observed for tumor necrosis factor receptor superfamily member 10A and 10B (TNFRSF10A, TNFRSF10B also named as TNF-related apoptosis-inducing ligand receptors TRAIL-R1 and TRAIL-R2, respectively), death effector domain containing 2 (DEDD2), inhibitor of kappa light polypeptide gene enhancer in B-cells, kinase gamma (IKBKG) and phosphoprotein enriched in astrocytes 15 (PEA15) genes.

## Discussion

There are several reports showing cytotoxic activity of *Phyllanthus* species [2, 20]. Some of them focus on lignans as the group of compounds responsible for the cytotoxic effect. However, most of the studies have concerned activity of raw *Phyllanthus* extracts and did not provide answers to the question which of the secondary metabolites are responsible for this biological activity [5, 21]. Recently Yu *et al*., isolated 33 compounds from the whole plant of *P*. *glaucus*, mainly lignans, phenylpropanoids and simple phenolics [5]. Among the isolated secondary metabolites of *P*. *glaucus* Yu *et al*. revealed that only one lignan—phyllanthusmin C belonging to arylnaphtalene lignan glycosides, showed cytotoxicity against HL-60, MCF-7 and SW480 cells [5]. By now there is no scientific evidence for cytotoxic activity of *Phyllanthus* species in conjunction with the presence of alkaloids.

In this study, we examined the effect of *P*. *glaucus* extract, alkaloid fraction and two main alkaloids—securinine and allosecurinine on HeLa cells. The tested compounds as well as the fraction demonstrated an antiproliferative activity on the cells in a dose-dependent and time-dependent manner. The results obtained by xCELLigence system and MTT assay indicated that securinine was significantly more active than allosecurinine, the fraction and extract. Furthermore, the effect of the alkaloid fraction was about 2-fold weaker than securinine and 2-fold stronger than allosecurinine. The differences in these potencies may result from differences in chemical structures of the two alkaloid compounds and their quantitative content in the fraction. The concentration of securinine in biomass was 3.58 mg/g d.w. while the content of allosecurinine was about 3 times lower (1.15 mg/g d.w.) [4].

Chemically, *Securinega*-type alkaloids belong to indolizidine alkaloids and are tetracyclic compounds with a 6-azabicyclo[3.2.1.]octane as the key structure to which an α,β-unsaturated-γ-lactone moiety and a piperidine ring are fused [6]. There are four naturally occurring stereoisomers of securinine which differ from each other with configuration of the ring in position 7-α, 9-α (securinine and allosecurinine, levo rotation) or 7-β, 9-β (virosecurinine and viroallosecurinine, dextro rotation) [22].

There are limited data comparing biological activity of securinine stereoisomers. In the study of enantiomers, it was stated that the pharmacological activity of virosecurinine [23] and allosecurinine [6] was weaker than L-securinine. It suggests that this activity is stereospecific.

Data concerning biological activity of allosecurinine are extremely limited—it was showed that allosecurinine inhibited spore germination of some saprophytic and pathogenic fungi [24]. In our work, the cytotoxic potential of allosecurinine was evaluated for the first time.

**Fig 11:**
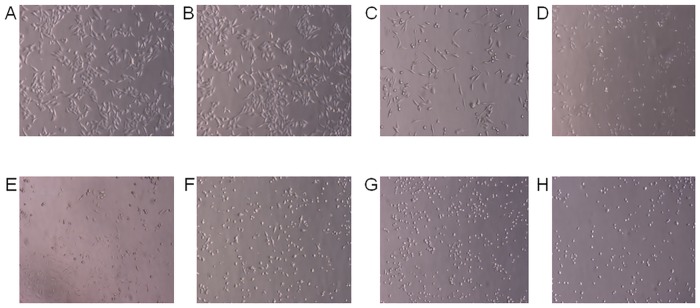
Microscopical examination of the effect of securinine on HeLa cells. The cells were treated with 0.5% DMSO (A) and the compound at concentrations of 1.0 (B), 5.0 (C), 10.0 (D), 15.0 (E), 20.0 (F), 30.0 (G), 50.0 μg/ml (H) for 24 h. The cellular changes were estimated in comparison to the control sample (DMSO).

Securinine, as the significantly potent antiproliferative compound, was investigated towards induction of HeLa cells apoptosis and evaluation of its action mechanism. Apoptosis is characterized by morphological and biochemical hallmarks, including cell shrinkage, membrane blebbing and condensation of nuclear chromatin [25]. These changes were observed in the tested cells treated with securinine (Figs 4 and 11).

Additionally, to confirm apoptosis proceeding in the cells incubated with this compound we determined the extent of plasma membrane asymmetry and loss of cellular membrane integrity by annexin. Annexin is a protein which binds to molecules of phosphatidylserine translocating from the inner to the outer surface of membrane of cells which undergo apoptosis [26]. We observed that the cellular apoptosis rate significantly increased in a dose-dependent manner. These results indicate that apoptosis is a major mechanism of induction of cell death by securinine.

One of the factors determining induction of apoptosis is oxidative stress and intracellular increase in production of superoxide radicals [27]. ROS can be generated as products of the mitochondrial oxidative phosphorylation process, or they may be produced during interaction with exogenous sources [27]. Oxidative stress occurs as the result of increased ROS production and insufficient ability of the cellular antioxidant system to defense. This leads to damage of proteins, nucleic acids, and lipids, and could promote cell apoptosis through cell cycle arrest and disruption of mitochondrial membrane integrity [27–30]. Furthermore, ROS play a role in regulating cellular events that lead to ERK (Extracellular signal-Related Kinase) activation which may contribute to cell death. ERK is involved in intrinsic pathway of apoptosis through acting on Bax and/or p53. Bax is translocated to the mitochondria and promotes the release of numerous pro-apoptotic proteins from the mitochondria to the cytosol [19, 31]. ERK may also regulate apoptosis and cell survival at other levels including TNF-α production, caspase-3, caspase-8 and Akt activities.

Our results show that the tested compound increased both ROS production and ERK1/2 activity. Furthermore, TNF receptors may be involved in ROS and ERK pathway and they may play a role as the agent-triggers of molecular signaling in the cells stimulated with securinine. During activation of cellular TNF receptors, oxidative stress may increase [32, 33] and lead to the cell cycle arrest that was observed in our study.

When the cell cycle is paused during oxidative stress, the ROS leads to the changes in mitochondrial membrane permeability and formation of pores in the membrane [29]. Permeability transition (PT) is always followed by the disruption of the mitochondrial membrane potential (MMP) and this effect is often observed in the early stages of apoptosis [34–36]. In our study, we observed that after treating HeLa cells with securinine the percentage of depolarized/live cells increased in a dose-dependent and time-dependent manner. This confirms the role of mitochondrial pathway in HeLa cell apoptosis induced by securinine.

Loss of the mitochondrial potential leads to the activation of caspase-9 and executioner caspases. The executioner caspases cleave various substrates including the nuclear and plasma membrane cytoskeletal proteins contributing to the biochemical and morphological changes observed in apoptotic cells [37, 38]. Our work shows that securinine changed the activity of initiator and effector caspases in HeLa cells undergoing apoptosis. The proposed pathway of securinine-induced cell death is presented in Figure B in S1 File.

The state of mitochondrial dysfunction and ROS generation as well as MAPK activation was not previously determined in cancer cells treated with *Securinega*-type alkaloids [7, 8, 10, 11]. Rana et al. revealed that securinine treatment leads to cell cycle arrest in G2/M phase in HCT 116 p53^+^ and p53^-^ cells. This study shows that the mechanism of the cell death in p53-deficient cells involves the induction of the proapoptotic protein and the p53 family member, p73 [10]. They also observed caspase-3/7 activation and morphological features of apoptosis. Xia et al. observed increase in number of autophagic SW480 cells treated with securinine and cell cycle arrest in G1 phase [11]. Cell cycle was also arrested in MCF-7 cells [7] and HL-60 cells [8] treated with securinine and SW480 cells incubated with virosecurinine [12]. In MCF-7 cells, securinine increased the expression levels of Bax and p53 which are well known as factors influencing on mitochondria and involved in ERK pathway [7]. Another study demonstrates that securinine induced apoptosis in HL-60 cells by modulation of the PI3K/AKT/mTOR pathway gene expression [8]. Securinine was also incubated with acute myeloid leukemia (AML) cell lines and primary patient samples. The compound led to cell differentiation with the activation of DNA damage signaling. Moreover, securinine demonstrated *in vivo* activity on AML in nude mice [39].

In conclusion, we compared the cytotoxic activity of *P*. *glaucus* extract, the alkaloid fraction and alkaloids—securinine and allosecurinine on HeLa cells. The most active tested compound—securinine exhibited antiproliferative effect and induced cell cycle arrest and mitochondrial apoptotic pathway. Securinine appears to be the compound with significant anticancer potential, however, the particular signal pathways of the cell death require further investigation especially as securinine could be potentially used in the treatment.

## Supporting Information

S1 File Apoptotic pathway induced by securinine from Phyllanthus glaucus in HeLa cells. **Figure A in S1 File. Securinine-induced changes in expression of genes in HeLa cells**. The cells were stimulated with securinine and DMSO (ctrl) at a concentration of 10.0 μg/ml and 0.1%, respectively and incubated for 24 h. The expression of genes was normalized to four endogenous control genes 18S, GAPDH, GUSB and HPRT1. The levels of expression of genes were generated by StepOne Software and they are presented as a fold-change over (a) or under (b) the value 1.0 (ctrl). **Figure B in S1 File. The proposed securinine-induced apoptotic pathway in HeLa cells. Table A in S1 File. Symbols of genes from TaqMan Array Human Apoptosis 96-well FAST Plates**.
(DOC)
