# Supplementary material for: Securinine from Phyllanthus glaucus Induces Cell Cycle Arrest and Apoptosis in Human Cervical Cancer HeLa Cells
Source: PLoS One. 2016 Oct 28;11(10):e0165372. doi: 10.1371/journal.pone.0165372 (PMC5085043; doi:10.1371/journal.pone.0165372)
Supplement: S1 File — Figure A in S1 File. Securinine-induced changes in expression of genes in HeLa cells. The cells were stimulated with securinine and DMSO (ctrl) at a concentration of 10.0 μg/ml and 0.1%, respectively and incubated for 24 h. The expression of genes was normalized to four endogenous control genes 18S, GAPDH, GUSB and HPRT1. The levels of expression of genes were generated by StepOne Software and they are presented as a fold-change over (a) or under (b) the value 1.0 (ctrl). Figure B in S1 File. The proposed securinine-induced apoptotic pathway in HeLa cells. Table A in S1 File. Symbols of genes from TaqMan Array Human Apoptosis 96-well FAST Plates. (DOC) [file pone.0165372.s001.doc]

Supporting Information

**Table A. Symbols of genes from TaqMan Array Human Apoptosis 96-well FAST Plates.**

| GENE SYMBOL | | | | | |
| --- | --- | --- | --- | --- | --- |
| 18S (control gene) | GAPDH (control gene) | HPRT1 (control gene) | GUSB (control gene) | BIRC2 | APAF1 |
| BCL2 | BCL2A1 | BCL2L1 | BCL2L10 | BCL2L11 | BCL2L13 |
| BIRC3 | XIAP | BIRC5,EPR1 | BIRC6 | BIRC7 | BIRC8 |
| CARD9 | CASP1 | CASP10 | CASP14 | CASP2 | CASP3 |
| CASP9 | CFLAR | CHUK | CRADD | DAPK1 | DEDD |
| HIP1 | HRK | HTRA2 | CARD18 | IKBKB | IKBKE |
| NFKB1 | NFKB2 | NFKBIA | NFKBIB | NFKBIE | NFKBIZ |
| RIPK1 | RIPK2 | TBK1 | TNF | TNFRSF10A | TNFRSF10B |
| BAD | BAK1 | BAX | BBC3 | BCAP31 | BCL10 |
| BCL2L14 | BCL2L2 | BCL3 | BID | BIK | NAIP |
| BNIP3 | BNIP3L | BOK | NOD2 | NOD1 | CARD6 |
| CASP4 | CASP5 | CASP6 | CASP7 | CASP8 | CASP8AP2 |
| DEDD2 | DIABLO | IFT57 | FADD | FAS | FASLG |
| IKBKG | LRDD | LTA | LTB | MCL1 | NLRP1 |
| PEA15 | PMAIP1 | PYCARD | REL | RELA | RELB |
| TNFRSF1A | TNFRSF1B | TNFRSF21 | TNFRSF25 | TNFSF10 | TRADD |


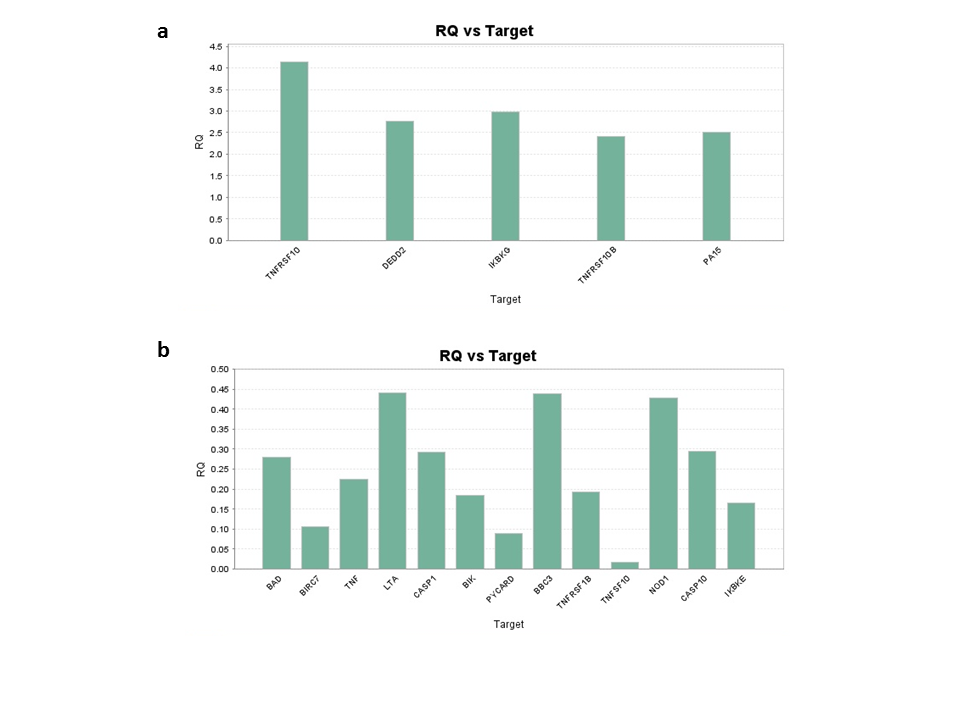


**Fig. A. Securinine-induced changes in expression of genes in HeLa cells**. The cells were stimulated with securinine and DMSO (ctrl) at a concentration of 10.0 µg/ml and 0.1%, respectively and incubated for 24 h. The expression of genes was normalized to four endogenous control genes 18S, GAPDH, GUSB and HPRT1. The levels of expression of genes were generated by StepOne Software and they are presented as a fold-change over (a) or under (b) the value 1.0 (ctrl).


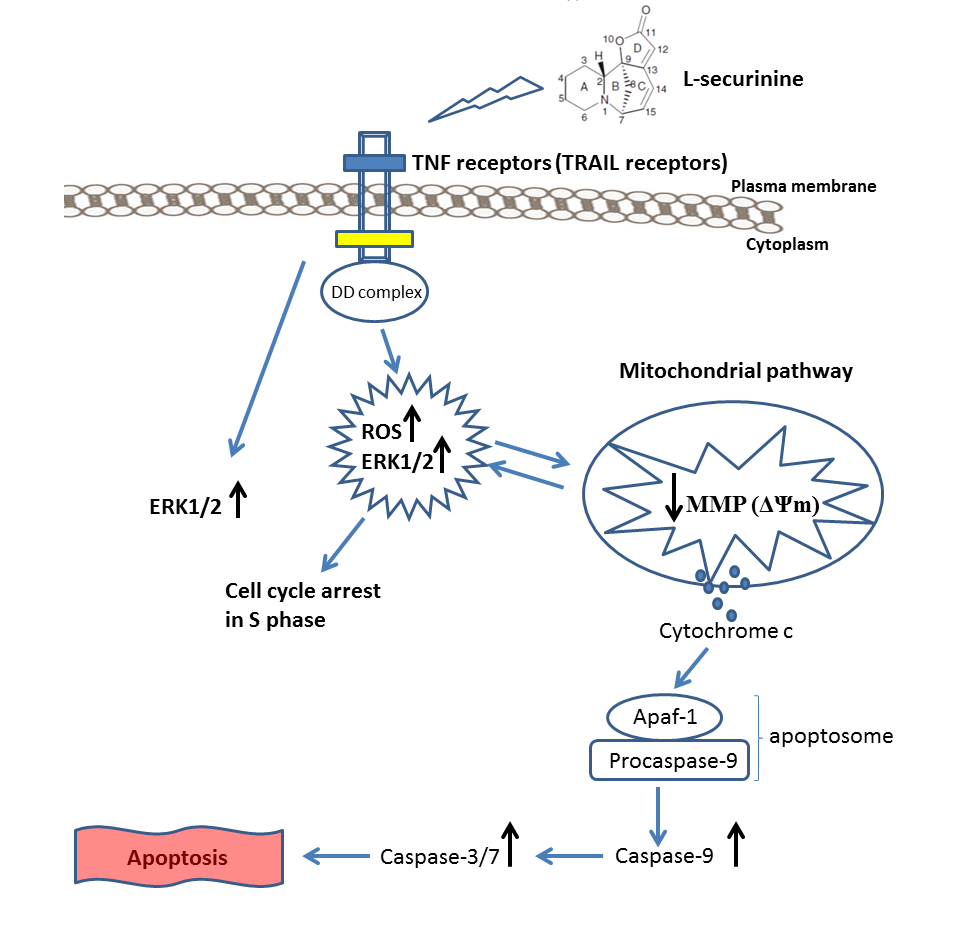


**Fig. B. The proposed securinine-induced apoptotic pathway in HeLa cells.**
